# Side-by-side placement of a novel slim 6-mm multi-hole covered self-expandable metallic stent for malignant hilar biliary obstruction

**DOI:** 10.1055/a-2569-7582

**Published:** 2025-04-15

**Authors:** Sho Takahashi, Toshio Fujisawa, Yusuke Takasaki, Daisuke Namima, Ko Tomishima, Shigeto Ishii, Hiroyuki Isayama

**Affiliations:** 1 Department of Gastroenterology, Jutendo University, Graduate School of Medicine, Tokyo, Japan

The drainage method for malignant hilar biliary obstruction (MHBO) has not been established. A 6-mm slim fully covered self-expandable metallic stent (FCSEMS) prevents tumor ingrowth and can be exchanged, but it carries the risk of segmental cholangitis by blocking the side branches of the bile duct 1 . A novel slim multi-hole FCSEMS (slim-MHSEMS) could resolve this issue 2 3 4 .

A 60-year-old man with pancreatic head cancer presented with Bismuth type IIIb MHBO ( Fig. 1 ). Bilateral stenting was performed using the slim-MHSEMS (HANARO Biliary Multi-hole Benefit; M.I. Tech Co., Ltd., Pyeongtaek, South Korea), which has six 1.6-mm holes evenly spaced along its circumference ( Fig. 2 ). The diameter of the delivery system of the slim-MHSEMS was 5.9-Fr, and simultaneous deployment was possible. Two slim-MHSEMSs (6 mm in diameter and 12 cm in length) were deployed simultaneously in side-by-side placement across the papilla ( Video 1 ). The tip of one slim-MHSEMS was placed in the B2 branch, and the contrast medium remained in B3 and B4 ( Fig. 3 ). However, it disappeared on an X-ray image taken the next day. Furthermore, the X-ray image revealed the pneumobilia of B4 ( Fig. 4 ).

**Fig. 1:**
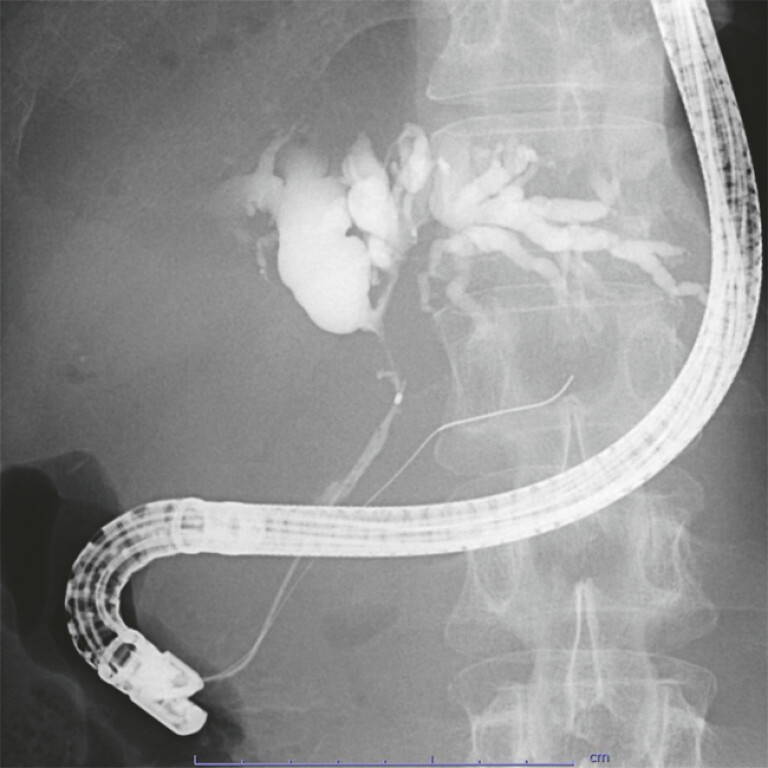
A fluoroscopic image presenting Bismuth type IIIb malignant hilar biliary obstruction.

**Fig. 2:**
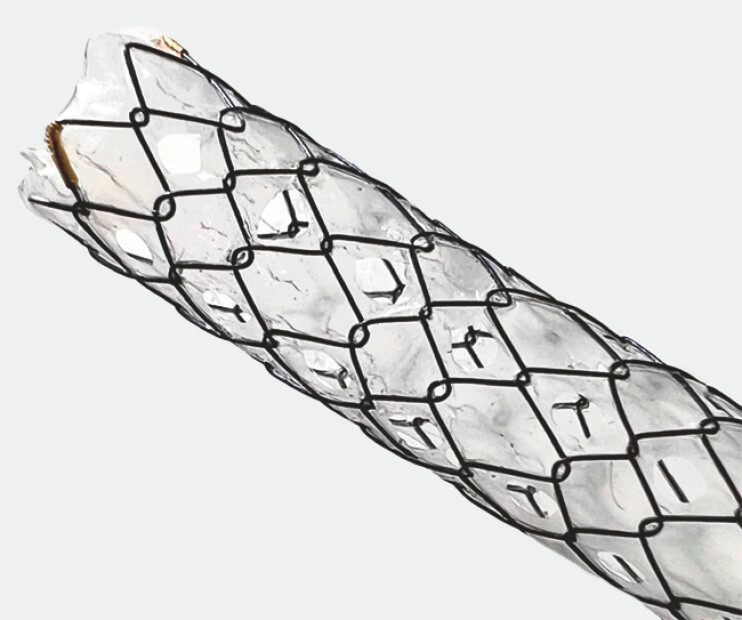
Enlarged image of the tip of the slim multi-hole self-expandable metallic stent (MHSEMS). It has six 1.6-mm holes evenly spaced along its circumference.

Two slim MHSEMSs (6 mm in diameter and 12 cm in length) were deployed simultaneously in side-by-side placement.Video 1

**Fig. 3:**
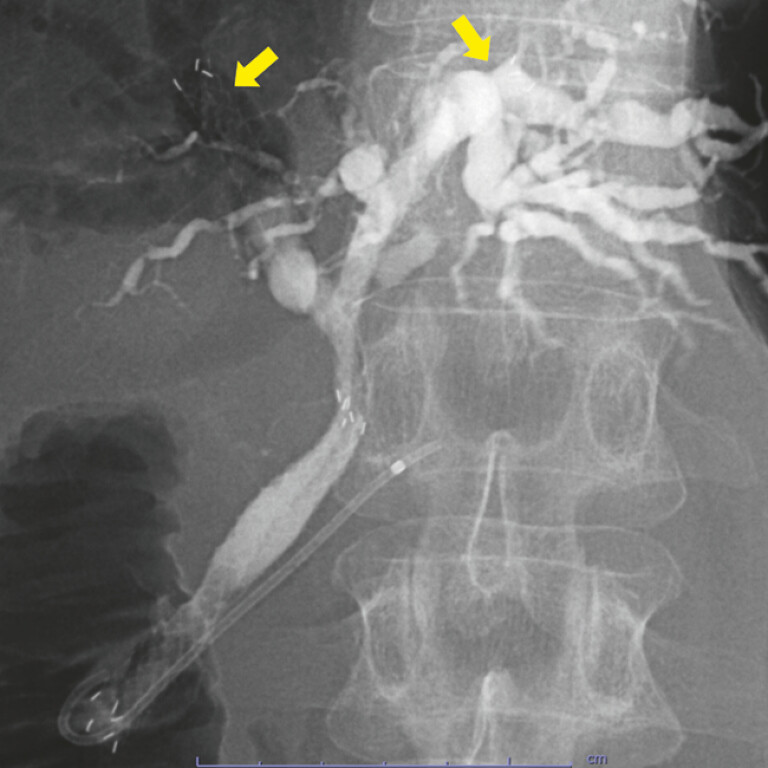
Slim-MHSEMSs were simultaneously deployed using side-by-side method. Yellow arrows: the proximal end of a slim MHSEMS in the B2 and B5 branches.

**Fig. 4:**
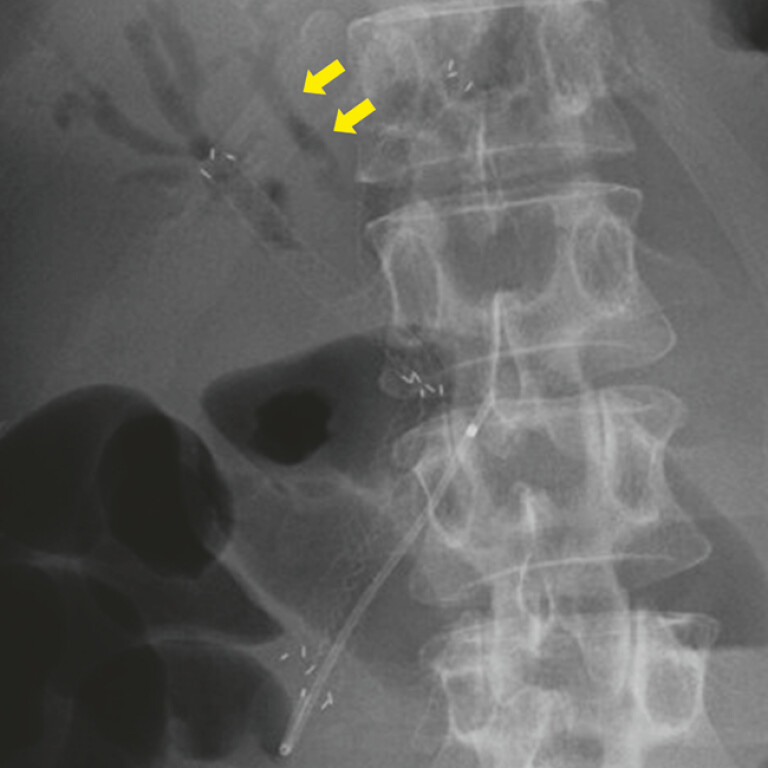
An X-ray image taken the next day reveals the disappearance of the contrast agent. Yellow arrows: pneumobilia in the B4 branch.

A fully covered SEMS has not been placed for MHBO due to the risk of segmental cholangitis. Because of the holes and diameter of the SEMS, the slim-MHSEMS can be safely and effectively placed for MHBO without obstructing the bile duct branches. Furthermore, simultaneous side-by-side placement of slim-MHSEMSs was easier than the previous bilateral stenting procedure. This report presents the first case of simultaneous side-by-side placement of slim-MHSEMSs for MHBO.

Endoscopy_UCTN_Code_TTT_1AR_2AZ

## Notes

Endoscopy E-Videos https://eref.thieme.de/e-videos
*E-Videos* is an open access online section of the journal *Endoscopy* , reporting on interesting cases and new techniques in gastroenterological endoscopy. All papers include a high-quality video and are published with a Creative Commons CC-BY license. Endoscopy E-Videos qualify for HINARI discounts and waivers and eligibility is automatically checked during the submission process. We grant 100% waivers to articles whose corresponding authors are based in Group A countries and 50% waivers to those who are based in Group B countries as classified by Research4Life (see: https://www.research4life.org/access/eligibility/ ). This section has its own submission website at https://mc.manuscriptcentral.com/e-videos .
